# “BEmotions” Intervention: A Pilot Study on Promoting Emotional Competence Among Emergency Department Nurses

**DOI:** 10.3390/healthcare14060715

**Published:** 2026-03-11

**Authors:** Vera Frazão, João Gomes, Filipe Batista, Tânia Morgado

**Affiliations:** 1 Hospital de Santo André, Unidade Local de Saúde da Região de Leiria, Rua de Santo André, 2410-197 Leiria, Portugal; vera.frazao@ulsrl.min-saude.pt (V.F.);; 2 School of Health Sciences, Polytechnic of Leiria, 2411-901 Leiria, Portugal; 3 Hospitais da Universidade de Coimbra, Unidade Local de Saúde de Coimbra, Praceta Professor Mota Pinto, Celas, 3004-561 Coimbra, Portugal; 4 Núcleo de Investigação da ULS Coimbra, Praceta Professor Mota Pinto, Celas, 3004-561 Coimbra, Portugal; 5 Health Sciences Research Unit: Nursing, University of Coimbra, Avenida Bissaya Barreto s/n, 3004-011 Coimbra, Portugal; 6 Nursing School of the University of Coimbra, University of Coimbra, Avenida Bissaya Barreto s/n, 3004-011 Coimbra, Portugal

**Keywords:** nurses, emotional intelligence, job satisfaction, emergency nursing, occupational health nursing, mental health, emotions

## Abstract

(1) **Background**: Working in emergency departments (EDs) affects healthcare professionals’ mental health and impacts their ability to provide safe, high-quality care. Emotional competence (EC) is recognized as a protective factor in occupational settings. The “BEmotions” intervention aimed to promote nurses’ EC and satisfaction with their work group in an ED in Portugal. (2) **Methods**: A quasi-experimental pilot study using a single-group pretest–posttest design was conducted. The BEmotions intervention was based on Veiga Branco’s construct of EC, which includes five dimensions: self-awareness, emotion management, self-motivation, empathy, and emotional regulation in groups. It consisted of five weekly sessions, each lasting 90 min. The instruments used were: (a) A self-developed sociodemographic questionnaire; (b) EVCE-r33—Veiga’s Reduced EC Scale; and (c) the Work Group Satisfaction Scale (ESAG). (3) **Results**: The study sample comprised 10 nurses, of whom 80% were female, with a mean age of 45.3 years (SD = 7.07). This pilot study suggests positive results regarding overall EC (*p* < 0.05; d = 0.81) and Job Satisfaction (JS) (*p* < 0.01; d = 1.22) among emergency department nurses. (4) **Conclusions**: the ‘BEmotions’ intervention suggests promising preliminary directions for replication in other contexts and for promoting mental health in the workplace; however, its methodological limitations highlight the need for further research.

## 1. Introduction

The concept of emotional competence (EC) initially emerged in the United States [1,2,3] and later in Spain [4], primarily in the contexts of education and training for children and adolescents. EC has been defined both as the demonstration of self-efficacy in social interactions that elicit emotional responses [1,2,3] and as the ability to effectively perform work-related tasks by mobilizing the requisite knowledge, skills, and understanding to achieve specific objectives [4]. EC is realized when individuals attain a desired level of personal fulfillment, which can be assessed through observable behaviors and attitudes or through self-reported reflections [5]. It is present across the lifespan and shapes how individuals express themselves and interact with their environment.

Studies on EC have examined diverse populations, including higher education instructors [6], primary school teachers [7,8], physiotherapists [9], social workers [10], Portuguese politicians [11], diabetic patients [12], parents of children with special needs [13], critical care health professionals [14], nursing students [15], and nurses across various hospital departments [16,17], as well as populations in gerontology [18], mental health and psychiatry [19,20,21], palliative care [22], and prison healthcare [23].

Understanding the development of EC is particularly crucial for health professionals whose practice involves providing care to individuals, families, groups, or communities [24]. Enhancing EC in health professionals not only safeguards their mental health but also supports the provision of safer and more effective care for critically ill patients [25].

In professions where interpersonal interactions are central, such as nursing, possessing knowledge and skills in interpersonal communication and EC is essential to deliver care aligned with a humanistic paradigm [26]. Nursing encompasses self-care, the cultivation of one’s inner world, and the nurturing of an internal environment, all in pursuit of holistic care for others [27].

EC significantly influences nurses’ attitudes by enhancing the use of effective coping strategies [28]. It contributes to emotional regulation, overall well-being, and resilience, thereby preventing stress, exhaustion, and burnout while supporting the delivery of high-quality, humanized care [29]. Developing EC is associated with enhanced conflict management, decision-making, communication, impulse control, emotional regulation, and persistence when facing challenges. These competencies contribute to a more balanced work environment and support clearer, goal-oriented clinical decision-making [30,31].

Emergency departments (EDs) are recognized as high-stress environments, requiring healthcare professionals to act quickly and accurately to provide timely support and comfort to patients and their families while managing life-threatening situations [32]. Several factors contribute to stress in EDs, including interpersonal conflicts, insufficient human resources, highly demanding routines to manage potentially traumatic situations, constant orders from supervisors, high workload, exposure to patient suffering and death, suicide, and direct or witnessed workplace violence [33].

ED nurses are exposed daily to a broader range of stressors and unpredictable working conditions compared to their peers in other settings. This heightened exposure places them at increased risk for depression, hypertension, anxiety, and elevated burnout levels, which can negatively impact nursing care quality, professional competencies, and work engagement [34].

A study in Morocco reported that nurses working in EDs experienced higher stress levels than those in general medical units [35]. Similarly, El Bouazzaoui et al. found that 32.4% of ED nurses exhibited high levels of emotional exhaustion [36].

Despite the evidence found regarding EC among nurses in other clinical settings, no studies were identified focusing specifically on nurses working in emergency care contexts. Comparable findings emerge when the term emotional intelligence (EI) is used, as it refers to the ability to recognize, understand, and manage one’s own emotions as well as those of others [37].

The current literature provides strong evidence supporting the relevance and effectiveness of EI training for nurses in high-intensity settings such as emergency and critical care units. Internationally, a quasi-experimental study in Egypt by Fattah et al. [38] demonstrated that structured EI programs significantly enhanced EI and reduced burnout among critical care nurses.

In Iran, a correlational study with mediation analysis by Alinejad et al. [39] showed that EI development mitigates occupational stress and improves nursing work quality, including in high-pressure emergency care contexts. Further Iranian research using structural equation modeling by Mehralian et al. [40] indicated that EI positively influences professional performance directly and indirectly through enhanced clinical competence. In another Iranian study [41], conducted with nurses with substantial experience in providing EI—based nursing care in intensive care, emergency, and medical units, the authors reported that by employing emotional skills and empathy, nurses enhanced the patient experience and improved the quality of nursing care.

Integrative and scoping reviews conducted in Portugal confirm that EI is a core professional competency among critical care nurses, with direct implications for psychological well-being, resilience, communication effectiveness, and patient safety. Collectively, these findings underscore the value of implementing structured EI training programs for nurses in emergency and critical care environments to enhance professional performance, decision-making, communication, and the capacity to handle complex clinical situations [42,43].

The complexity of healthcare demands that professionals mobilize diverse competencies, including EC, which, alongside skills such as empathy, resilience, social support, and job satisfaction (JS), serves as a protective factor under high-stress conditions [25].

Traditionally, JS has been defined as a positive emotional response arising from an individual’s evaluation of their work or work experiences [44]. In team contexts, JS reflects members’ affective responses toward the group, including task accomplishment and the group’s affective climate [45]. JS is a key indicator of healthcare quality due to its relevance for employee well-being and performance.

Nurses’ salaries are the most frequently cited factor contributing to job dissatisfaction, along with unmet safety needs related to episodes of violence in emergency care settings, government budget cuts in the health sector, work-related stress, and difficulties in achieving an adequate work–life balance [46]. Extant research demonstrates the direct and significant impact of EI on nurses’ JS [47,48,49,50,51,52,53,54,55]. A systematic review conducted with nurses working in intensive care units [50] revealed that, following EI training, participants exhibited increased levels not only of EI but also of JS.

Emergency department nurses are frequently exposed to unpredictable working conditions and high levels of occupational stress, placing them at increased risk of emotional exhaustion, reduced JS, and diminished quality of care. Extant research shows that EC is a critical factor in promoting psychological well-being, effective communication, and safe clinical practice. However, no structured EC programs specifically designed for emergency nurses have been identified.

Given this gap, a pilot study is required to examine the feasibility, acceptability, and preliminary effects of an intervention targeting EC in this population. The “BEmotions” program was therefore developed to enhance emergency nurses’ EC with potential benefits for JS and team functioning.

This pilot study aims to explore the preliminary impact of the “BEmotions” intervention on nurses’ EC and JS, thereby providing insights to inform the design of a larger trial.

## 2. Materials and Methods

### 2.1. Study Design

A quasi-experimental pilot study was carried out in central Portugal, utilizing a one-group pretest–posttest design, in which participants were assessed before and after the intervention without the inclusion of a control group.

The “BEmotions” intervention comprised five weekly 90 min sessions, conducted in a closed-group format in the afternoon, in a room provided by the health institution. Each session focused on one of the domains of emotional EC according to the construct proposed by Veiga-Branco: self-awareness, emotion management, self-motivation, empathy, and group emotion management [7].

An experiential methodology was employed, in which learning is developed through reflection on practical experiences. The way these experiences are perceived is influenced by both internal (self-related) and external (contextual) components. The approach was grounded in cognitive-behavioral principles, emphasizing the role of thought in shaping emotions and how these, in turn, influence behavior and effective problem-solving [56]. To foster EC development, a practical methodology was applied, incorporating group dynamics, the use of technology (such as videos and mobile applications), sociodramatic techniques (sociometric action, tableaux, and dramatic play), breathing and relaxation exercises, mindfulness practices, reminiscence, role-playing, brainstorming, and debriefing activities.

Each session began with a summary and systematization of the conclusions from the previous session and concluded with a synthesis of key ideas discussed, along with the assignment of homework tasks for participants to complete between sessions.

The sessions were held by a nurse, within the scope of the master’s degree and specialty in mental health nursing and psychiatry under the supervision and guidance of a nurse specialist in nursing specialty of mental health and psychiatry.

The content of the sessions may be transferable to all healthcare professionals. Although the intervention was originally designed for nurses, it can be adapted to other clinical contexts through the inclusion of profession-specific practical examples.

As a motivational strategy for the implementation of the “BEmotions” intervention, a bus journey metaphor was employed to symbolize the alignment and progression of the intervention sessions. This journey was introduced to the nurses through a bookmark featuring an illustrative itinerary, serving simultaneously as an invitation and a visual reminder.

The five planned sessions, described in Supplementary Materials, were conceptualized as tourist destinations, each corresponding to different emotional competencies, with activities designed in accordance with each “location” to be explored. To reinforce this approach, an infographic was displayed in the workplace.

Drawing inspiration from Eduardo Lourenço’s quotation, “More important than the destination is the journey” (LOURENÇO, 2009) [57], the intervention encourages professionals to value the emotional and relational processes experienced within the team, emphasizing that personal and professional growth occurs throughout the shared journey, rather than solely at the final goal.

### 2.2. Participants

The population consisted of all nurses working in the ED of a hospital in the Central Region of Portugal. Nurses in management positions, on sick leave, on vacation, or those who refused to participate were excluded. The sample was non-probabilistic and intentional, comprising 10 nurses (50% of the population) who agreed to participate and attended at least three of the five planned sessions, given that this was a criterion previously defined by the investigators, in the absence of a standardized benchmark. Specifically, four nurses attended all five sessions, four attended four sessions, and two attended only three of the five sessions.

### 2.3. Data Collection Instruments

A two-part questionnaire was administered. The first part included questions regarding participants’ sociodemographic characteristics (gender, age, marital status, educational qualifications) and professional characteristics (years of professional experience, length of time working with the team, type of employment contract, specialist title granted by the Portuguese Order of Nurses and respective area of specialization, and any training in EI or EC).

The second part included two data collection instruments validated for European Portuguese. A detailed description of the instruments, including dimensions, number of items, response scales, reverse-coded items and scoring procedures, is provided in Table 1.

The Veiga Scale of EC—Reduced Version (EVCE-r33) [58] demonstrates high reliability, with an overall Cronbach’s alpha coefficient of 0.942. In the present study, the EVCE-r33 showed good overall internal consistency, with Cronbach’s alpha values of 0.930 at the pre-intervention assessment and 0.957 at the post-intervention assessment. Across its subscales, Cronbach’s alpha coefficients were also adequate, with values exceeding 0.70 at both measurement points.

The Satisfaction with the Work Group (ESAG) instrument [45] has previously demonstrated good internal consistency, with a Cronbach’s alpha of 0.910. In the current study, the ESAG proved to be a reliable and consistent instrument for assessing satisfaction in group work contexts, yielding Cronbach’s alpha values of 0.727 at the pre-intervention assessment and 0.852 at the post-intervention assessment.

### 2.4. Data Collection

This study was carried out in 2025, with data collected in January and February, both prior to and following the “BEmotions” intervention. All data, gathered via the institutional Google Forms platform.

### 2.5. Data Analysis

The Statistical Package of Social Science (SPSS) version 26.0 for Windows^®^ and the G*Power software version 3.1.9.7, were used to carry out the statistical analysis.

Descriptive statistics techniques were used, including frequencies (absolute and relative), measures of central tendency (mean and median), measures of dispersion (variance, standard deviation, interquartile range, range, minimum and maximum values), and measures of skewness and kurtosis (skewness coefficient and kurtosis coefficient).

To assess the distribution shape, skewness and kurtosis coefficients were calculated, along with the identification of potential outliers. The normality of the distributions was tested for each variable in the samples using the Shapiro–Wilk test, appropriate for small sample sizes (n < 30).

For the inferential analysis, the assumptions underlying each statistical test were verified prior to their application. A range of statistical procedures was employed to test the research hypotheses. Hypotheses 1 and 2 were tested using the parametric Student’s *t*-test for paired samples, while Hypothesis 3 was tested using the non-parametric Wilcoxon signed-rank test for paired samples.

The calculation of effect size and test power for the hypotheses was performed using G*Power software. Since the hypothesis tests belong to the d family (which includes measures estimating the magnitude of the difference between means), we followed Cohen’s guidelines for interpreting effect sizes: d = 0.20—small effect; d = 0.50—medium effect; d = 0.80—large effect [59].

A significance level of 0.05 was adopted for the hypothesis tests, as this is the standard threshold commonly used in social sciences research [60,61,62,63].

### 2.6. Ethical Procedures

This study was approved by the Ethics Committee of the Local Health Unit of the Leiria region (No. 48/2024). All potential participants were informed of the objectives of this study prior to data collection and could withdraw from this study without any repercussions. The ethical principles outlined in the revised Declaration of Helsinki [64] were followed, and all participants provided informed consent, which emphasized voluntary participation and the ethical safeguards of confidentiality.

## 3. Results

### 3.1. Sociodemographic and Professional Characterization of the Sample

It was found that most of the participants were female 8 (80%), with an average age of 45.3 years (SD: 7.07; Xmin–Xmax: 38–59), 9 (90%) are married/married in a de facto union and 1 (10%) divorced/single, 9 (90%) have a bachelor’s degree and 1 (10%) has a master’s degree.

Related to professional experience, 2 (20%) have between 5 and 15 years, 5 (50%) have between 16 and 25 years, and 3 (30%) have more than 25 years. Length of time working in the team: 4 (40%) members have been working for up to 10 years, 4 (40%) between 11 and 20 years and 2 (20%) more than 20 years. 5 (50%) members have an open-ended contract and 5 (50%) have a public service contract. 1 (10%) holds a specialist title from the OE in community nursing and 1 (10%) of the participants has training in the area of EI/EC.

### 3.2. Hypothesis 1—There Is a Significant Difference in Nurses’ EC After the “BEmotions” Intervention

Having verified the conditions for its application, we used the *t*-test for two paired samples to test this hypothesis. At a significance level of 0.05, there is evidence to affirm that the average global emotional competencies are statistically different between the pre- and post-intervention moments (t_(9)_ = 2.481; *p* < 0.05).

In fact, the nurses’ EC was higher post-intervention ($\bar{x}$ = 5.01 ± 0.83) than pre-intervention ($\bar{x}$ = 4.79 ± 0.69) (Table 2).

In terms of effect size, the dimensions empathy and emotional management in groups had a magnitude considered large, with the caveat that the power is low, which increases the likelihood of making a type II error (false negative). The effect size for the self-awareness and emotion management dimensions is medium, but their power is very low. The self-motivation dimension was the one where we found the smallest effect size and negligible potency, with the two distribution curves practically overlapping (Table 3).

Based on these results, it can be concluded that the “BEmotions” intervention had positive effects on increasing nurses’ overall. Although the differences were not statistically significant, the dimensions empathy and emotional management in groups had a large magnitude of effect, an inference limited by the small sample size.

### 3.3. Hypothesis 2—There Is a Significant Difference in Nurses’ Satisfaction with the Work Group After the “BEmotions” Intervention

The nurses’ satisfaction with the work group in this sample belong was higher at the post-intervention moment ($\bar{x}$ = 5.39 ± 0.45) when compared to the Baseline moment ($\bar{x}$ = 5.00 ± 0.48) (Table 4).

A large effect size and high statistical power were obtained (d = 1.22; α = 0.05; π (1 − β) = 0.93). This value indicates that the difference between the pre- and post-intervention mean scores (M_dif_ = 0.39) is 1.22 standard deviation units of the difference scores (SD_dif_ = 0.32), which is considered a large effect magnitude according to Cohen’s classification [59]. Based on these results, we can infer that the intervention program had a large effect on the nurses’satisfaction with the work group.

### 3.4. Hypothesis 3—There Is a Significant Difference in the Overall Satisfaction with the Work Group of the Nurses After the “BEmotions” Intervention

The results suggest that the “BEmotions” intervention increased nurses’ overall satisfaction with the work group (Wilcoxon Z = −2.264; *p* < 0.05). It was found that no nurse’s overall satisfaction with the work group rating decreased, six nurses improved and four nuses maintained the same overall satisfaction with the work group after the “BEmotions” intervention (Table 5).

The magnitude of the effect was found to be large (d_z_ = 0.98; α = 0.05; π (1 − β) = 0.71), according to Cohen’s classification [59].

The results suggest that nurses’ overall satisfaction with the work group improved following their participation in the “BEmotions” intervention (Table 6).

## 4. Discussion

Although the preliminary results from the implementation of the “BEmotions” intervention are promising with regard to enhancing ED nurses’ overall EC and align with findings from other studies involving nurses in different contexts [17,20,22,23,55,64], further in-depth investigation is warranted due to the lack of statistical significance and limitations associated with the small sample size.

Nurses demonstrated a moderate overall EC profile [17,20,22,23,61,65]. The complex and demanding nature of the professional context in which nurses operate requires constant emotional regulation, empathy, and effective communication skills, particularly in high-stress environments such as emergency departments, intensive care units, and other clinically challenging settings. These conditions foster the development of specific dimensions of EC. Although a moderate overall EC profile among these nurses reflects a balanced level, it could be further enhanced through programs designed to develop and consolidate higher levels of EC in these professionals.

Self-awareness being the most developed dimension [20,22,23,55,65,66,67,68]. This result may be explained by the specific characteristics of the nursing profession and the reflective nature of caring. Nursing practice allows professionals to effectively interconnect emotions, thoughts, and behaviors, which becomes essential for decision-making and creativity, often guided by feelings or intuition [69].

Nursing education, grounded in a holistic and person-centred approach, fosters the development of self-awareness from the earliest stages of academic training through reflective practice, clinical supervision, and the analysis of personal emotional experiences. Furthermore, the direct and continuous contact with patients’ suffering, pain, and vulnerability enhances the need for self-knowledge and emotional balance as a means of preventing professional burnout and ensuring the quality of care.

Emotion management exhibited the largest mean difference, while self-motivation showed the smallest. Effect size analysis indicated large effects for empathy and group emotion management, whereas self-awareness and emotion management showed medium effects. Self-motivation demonstrated negligible effect and almost complete overlap of pre- and post-intervention distributions, this reinforces the need to continue investing in emotional education content.

Regarding nurses’ satisfaction with their work group, scores increased from baseline to post-intervention, indicating a positive effect. What most influenced satisfaction with the work group were the items related to the leader: “How the leader organizes and coordinates the team’s activities”, and “Relationships between team members and the leader”, corroborating the findings of other authors [70,71,72]. It should be noted that head nurses play an important role in establishing strategies that can improve the working environment and promote nurses’ JS [73]. They are fundamental not only in the management of human and material resources, but also in the effectiveness of communication and relationships established between professionals, particularly with regard to conflict management [71]. In the study by Rocha et al., a statistically significant decrease in nurses’ JS with regard to their hierarchical superior was observed following the implementation of the SafeCare Model [67]. These results were attributed to a change in the head nurse during the intervention, thereby highlighting the importance of leadership in JS.

Followed by “Relationships between team members”, corroborating other studies [70,72]. It is through the relationships established at work that individuals can feel socially and affectively supported, and in this sense contribute to work health [74].

Furthermore, overall satisfaction with the work group improved suggesting that the “BEmotions” intervention had a positive impact on nurses’ perceptions of their work environment.

With regard to the “BEmotions” intervention, several strengths were noted, including participant motivation, the integration of the sessions into the nurses’ in-service training plan, and the appropriateness of the format, duration, and strategies employed.

Nonetheless, important limitations were identified, such as the small sample size, the single-center design, the absence of a control group, the lack of randomization, and the fact that the sample size formula was calculated retrospectively. These limitations, together with the absence of follow-up monitoring, restrict the capacity to robustly assess the effectiveness of this type of intervention. Given these limitations, this study is considered a pilot study, a small-scale investigation designed to assess the primary outcomes of a future large-scale study [75,76]. Future research should include experimental studies with larger samples to assess the effectiveness of this kind of intervention.

### Implications for Practice, Education and Research

The “BEmotions” intervention has implications for nursing pratice and to the Promotion of Mental Health and Well-being in the workplace. Since EI has been shown to positively influence the biopsychosocial well-being of health professionals, increasing their resilience, their perception of social support, empathy, their performance and JS, reducing stress [77]. Since the late 1990s, the WHO considers EI one of the ten life skills that support people to act adaptively and positively [78].

In view of the above, and in order for projects in the CE and TS scope to be implemented, it is urgent that institutional decision-makers provide conditions for their execution in a work context, namely the inclusion of these projects in the scope of occupational health. It is suggested that mental health professionals, including mental health and psychiatric specialist nurses, be integrated as dynamic facilitators of the “BEmotions” intervention, and that the intervention be replicated with other nursing teams and/or groups of healthcare professionals.

From a global perspective of the higher education system, namely related to academic courses in the area of health, the development of CE may bring challenges towards the integration of these contents in the curricular plans of nurses and/or other health professionals. Veiga-Branco [8] proposes the inclusion of emotional education as a cross-cutting theme throughout the entire training and curricular path for the intra and interpersonal development of future health professionals in general and nursing professionals in particular.

From the perspective of mental health nursing, it has implications for the academic training of the mental health nurses, since in their clinical practice it is the professional who mobilizes himself as a therapeutic instrument in relational excellence [78,79] and must know himself, have a perception of himself and what he feels, during his intervention. And he must be able to read his own frame of consciousness, relating to emotional phenomena already experienced, in addition to the ability to manage his behavior and the perception of what he shows to others, whether they are his users or teammates [8].

Future research should include experimental studies with larger samples to assess the effectiveness of this kind of intervention.

## 5. Conclusions

According to the collected results, the “BEmotions” intervention appears to be both feasible and acceptable. This pilot study suggests a promising direction, demonstrating the effectiveness of the “BEmotions” intervention in promoting EC among emergency department nurses in relation to JS. However, several methodological limitations have been identified, highlighting the need for further research.

The development of the “BEmotions” intervention, aimed at improving the quality of care by promoting EC and ST, provided an opportunity to raise awareness among both the sample and the institution on this topic, creating space for future studies to be conducted.

There remains a clear need for further in-depth research on EC among nurses providing care in emergency contexts, particularly to clarify how EC contributes to critical outcomes in the ED environment, including emergency decision-making, team communication, and the safe management of critically ill patients.

The promotion of EC among healthcare professionals has important implications for nurses’ mental health and workplace well-being, ultimately contributing to the delivery of high-quality and safe patient care.

## Figures and Tables

**Table 1:** Detailed description of the instruments EVCE-r33 and ESAG.

Instrument	Dimensions	Number of Items	Response Scale	Reverse-Coded Items	Scoring
**EVCE-r33** (Veiga Scale of EC—Reduced Version) [58]	Self-awareness (8 items); Emotion management (7 items); Self-motivation (7 items); Empathy (5 items); Emotion management in groups (6 items)	33	7-point Likert (1 = ‘never’, 2 = ‘rarely’, 3 = ‘infrequently’, 4 = ‘usually’, 5 = ‘frequently’, 6 = ‘very frequently’, and 7 = ‘always’)	self-awareness (items a, c, d, e, f, g, h); emotion management (items c, d, e, g), self-motivation (items a, d, e, f, g), and empathy (item e)	Dimension scores: mean of the items within each dimension. Global Emotional Competence score: mean of the five dimension means;
**Levels:** low (1.00–3.49), moderate (3.50–5.45), and high (5.46–7.00)					
**ESAG** (Satisfaction with the Work Group) [45]	satisfaction with affective aspects, such as interpersonal relationships among team members (3 items); assess satisfaction with the task system, including how the team works and the roles performed by each member (4 items)	7	7-point Likert (1 = totally dissatisfied, 2 = very dissatisfied, 3 = moderately dissatisfied, 4 = neither satisfied nor dissatisfied, 5 = moderately satisfied, 6 = very satisfied, 7 = totally satisfied)	none	Mean score

**Table 2:** Descriptive measures of the EVCE-r33 (pre-intervention/post-intervention).

Dimensions and Overall	Pre-Intervention				Post-Intervention			
	$\bar{x}$	SD	Min	Max	$\bar{x}$	SD	Min	Max
Self-awareness	5.29	0.87	3.50	6.25	5.51	1.04	3.00	6.50
Emotion Management	4.61	0.85	2.43	5.29	4.87	1.12	1.86	5.86
Self-motivation	4.99	1.10	2.57	6.14	5.10	1.00	3.00	6.43
Empathy	4.94	0.81	4.00	6.20	5.18	0.69	4.20	6.20
Emotional Management in Groups	3.98	0.76	2.50	5.33	4.22	0.69	3.33	5.33
Global EC	4.79	0.69	3.24	5.61	5.01	0.83	3.15	6.09

**Table 3:** Effect size and power of the Global EVCE-r33 and its dimensions.

Dimensions and Overall	Effect Size d_z_	Power π (1 − *β*)	Classification Effect Size
Self-awareness	0.51	0.30	Medium
Emotion Management	0.57	0.37	Medium
Self-motivation	0.20	0.09	Small
Empathy	0.90	0.72	Large
Relationship Management	0.82	0.63	Large
Global EC	0.81	0.62	Large

**Table 4:** Descriptive measures (pre-intervention/post-intervention).

Items	Pre-Intervention					Post-Intervention				
	$\bar{x}$	Md	SD	Min	Max	$\bar{x}$	Md	SD	Min	Max
1. Existing climate in the work team.	4.90	5.00	0.88	3	6	5.40	5.00	0.52	5	6
2. The team’s way of working.	4.60	5.00	0.70	3	5	5.10	5.00	0.57	4	6
3. How the leader organizes and coordinates the team’s activities.	5.20	5.00	0.63	4	6	5.50	5.50	0.85	4	7
4. Results achieved by the work team.	5.10	5.00	0.32	5	6	5.40	5.00	0.52	5	6
5. Relationships between team members and the leader.	5.50	5.50	0.53	5	6	5.70	6.00	0.67	5	7
6. Relationships between the members of the work team.	5.00	5.00	1.05	3	6	5.40	5.00	0.52	5	6
7. Role that each member plays in the team.	4.70	5.00	1.06	3	6	5.20	5.00	0.63	4	6
Global satisfaction with the work group	5.00	5.07	0.48	4.43	5.71	5.39	5.21	0.45	5.00	6.14

**Table 5:** Descriptive measures and Wilcoxon test for two paired samples of overall satisfaction with the work group.

Overall Satisfaction with the Work Group		Description		Test Statistics	
		N	MR_k_	Z	Sig.
Pre-Intervention	Negative Posts	6 ^a^	3.50	−2.264 ^d^	0.024
–	Positive Posts	0 ^b^	0.00		
Post-Intervention	Draws	4 ^c^	-		

^a^ Overall satisfaction with the work group before < overall satisfaction with the work group after. ^b^ Overall satisfaction with the work group before > overall satisfaction with the work group after. ^c^ Overall satisfaction with the work group before = overall satisfaction with the work group after. ^d^ Based on positive posts.

**Table 6:** Descriptive measures of overall satisfaction with the work group.

Overall Satisfaction with the Work Group	$\bar{x}$	SD	Md	AIQ	Min	Máx
Pre-Intervention	7.30	0.82	7.50	1.00	6	8
Post-Intervention	8.20	1.14	8.00	2.00	7	10

## Data Availability

The original contributions presented in this study are included in the article/Supplementary Materials. Further inquiries can be directed to the corresponding author.

## Supplementary Materials

The following supporting information can be downloaded at: https://www.mdpi.com/article/10.3390/healthcare14060715/s1, Table S1. Session plan 1—Alcobaça—“Self-awareness”. Table S2. Session plan 2—Porto—“Management of emotions”. Table S3. Session plan 3—Coimbra—“Self-motivation”. Table S4. Session plan 4—Lisbon—“Empathy”. Table S5. Session plan 5—Algarve—“Emotional management in groups”.
