# Supplementary material for: “BEmotions” Intervention: A Pilot Study on Promoting Emotional Competence Among Emergency Department Nurses
Source: Healthcare (Basel). 2026 Mar 11;14(6):715. doi: 10.3390/healthcare14060715 (PMC13027072; doi:10.3390/healthcare14060715)
Supplement: Supplementary file 1 [file healthcare-14-00715-s001.zip › healthcare-3988931-supplementary.pdf]

Supplementary Materials

Table S1. Session plan 1 - Alcobaça – "Self-awareness"

**Session 1**	**Alcobaça – "Self-awareness"**
**Duration**	90 minutes
**Objectives**	Present the psychoeducational intervention to the nursing team;<br>Fill in the written informed consent and assessment instruments;<br>Develop emotional self-awareness through the identification and recognition of emotions;<br>Increase Emotional Literacy;<br>Increase satisfaction with the work group.
**Materials and Media**	Large room, computer, projector, hats of different colors, invitation "travel itinerary" and informed consent form;
**Strategies/Activities**	
**Introduction**	Contextualization of the origin and relevance of the psychoeducational intervention *Bemotions:* concepts of EI and CE, highlighting their conceptual differentiation, as well as the identification of the problem related to WH and its correlation with EC.
**Development**	Expository method:<br>Presentation of the psychoeducational intervention *BEmotions*;<br>Delivery of the "Travel Itinerary" invitation

Session 1	Alcobaça – "Self-awareness"
	Fill:<br>Informed consent;<br>Assessment instruments: Sociodemographic Questionnaire, Veiga Emotional Competence Scale – short version (EVCEr33) (VEIGA-BRANCO, 2023), Satisfaction with the Working Group Scale (DIMAS, 2018).<br><br>Icebreaker "The best and most important source of self-care is the relationship that professionals establish with each other" (NUNES, 2024): arrange the participants in a circle, ask them to write on a balloon characteristics and qualities of the colleague on the right, launch the balloons into the air without letting them fall. By randomly picking up the balloons, participants must guess who they belong to.<br><br>Expository method:<br>Concept of emotion;<br>Emotion VS feeling;<br>Functions of emotions;<br>Basic emotions: sadness, joy, anger, disgust, fear.<br><br>"Me and my emotions" dynamic<br>1. Arrange the participants sitting in a circle;<br>Ask each participant to take turns putting on the various hats (each color of the hat corresponds to an emotion: sadness, joy, fear, anger, love, hope) naming what they feel "I feel (emotion), when..."<br><br>2. Each participant must choose a hat related to an emotion, individually explaining the reason for the choice. Finally, ask the participants to make a "statue" that reflects this emotion through the body, in a group or individually according to the chosen emotion;

Session 1	Alcobaça – "Self-awareness"
Conclusion	Gather participants in a semicircle and invite them to share how they felt during the activity and what they learned about emotional self-awareness. Summary and return of ideas.<br><br>Completion of the Satisfaction with Session 1 questionnaire.
Work to think	During the next week, identify a situation that has triggered an emotion (which) and what its physiological manifestation was.

Fonte: Elaborate Investigators (2025)

Table S2. Session plan 2 - Porto – "Management of emotions"

Session 2	Porto – "Management of emotions"
Duration	90 minutos
Objectives	To learn about the concept of emotional self-regulation;<br>Learn about long-term and immediate emotional self-regulation strategies;<br>Increase satisfaction with the work group;<br>Increase satisfaction with the work group.

Session 2	Porto – "Management of emotions"
Materials and Media	Computer, music speaker, relaxation script
	Strategies/Activities
Introduction	Summarize previous session;<br>Joint discussion on the TPP;<br>Introduce a new theme with *brainstorming* on emotion management;
Development	Expository Method:<br>Concept of emotional self-regulation;<br>*Brainstorming* about a fictitious situation;<br>Long-term emotional self-regulation strategies;<br>Strategies of emotional self-regulation in the immediate (Modal Model).<br><br>Reminiscence through team photographs;<br>Diaphragmatic breathing training;<br>Meditação do chocolate (Mindfulness eating),<br>Relaxation session by guided imagination.
Conclusion	Summary and feedback of ideas;<br>Completion of the Satisfaction with Session 2 questionnaire.
Work to think	Bring an object/prop/message, etc. to the next session in order to build a "survival box- Emotion management" taking into account an emotional management strategy.

Session 2	Porto – "Management of emotions"

Fonte: Elaborate Investigators (2025)

Table S3. Session plan 3 - Coimbra – "Self-motivation"

Session 3	Coimbra – "Self-motivation"
**Duration**	90 minutes
**Objectives**	Learn about the concept of motivation and self-motivation;<br>Learn to set realistic achievable goals;<br>Increase satisfaction with the work group.
**Materials and Media**	Printed "Wheel of Life", pens
**Strategies/Activities**	
**Introduction**	Summarize the previous session;<br>Reflection on TPP from session 2;<br>Introduce a new topic with *brainstorming* on Self-motivation;

Session 3	Coimbra – "Self-motivation"
**Development**	Active Method:<br>What motivates you? (*Mentimeter*);<br>Myths about motivation (*Kahoot!);*<br><br>Expository Method:<br>Concept of motivation;<br>Types of motivation;<br>Self-motivation concept;<br>The influence of emotions (optimism, hope and anxiety) on motivation;<br>Concept of resilience;<br>How to set realistic goals through the SMART mnemonics.<br><br>Presentation and completion of the "Wheel of Life":<br>1. Ask participants to observe the wheel of life that includes 4 main areas: professional, relationships, personal and quality of life. For each area, participants must paint the value that corresponds to their satisfaction at the moment;<br>2. Ask participants to observe which areas obtained the lowest score by reflecting on what needs to happen, what they would like to do or have to increase this value;<br>3. Of the areas that did not obtain a score of 10, which ones would you like to invest in at this time?.
**Conclusion**	Summary and return of ideas: "More important than the destination, is the trip.";<br>Completion of the satisfaction questionnaire for session 3.
**Work to think**	Distribute a sheet of paper to each participant with the four domains of the wheel of life (Personal, Relationships, Professional, Quality of life) and ask them to write down for each one a goal they would like to achieve through the SMART methodology (Specific, Measurable, Attainable; relevant, temporal);

Session 3	Coimbra – "Self-motivation"
	Develop a detailed action plan: steps, resources, possible obstacles and how to overcome them, and deadlines; (encourage participants to be realistic and flexible);<br>Sharing plans in a group (optional).

Fonte: Elaborate Investigators (2025)

Table S4. Session plan 4 - Lisbon – "Empathy"

Session 4	Lisbon – "Empathy"
**Duration**	90 minutes
**Objectives**	Learn about the concept of Empathy;<br>Increase empathy between participants by promoting understanding of the perspectives and experiences of others;<br>Increase teamwork;<br>Promote flexibility and tolerance;<br>Promote acceptance of difference;<br>Encourage respect;<br>Reduce stereotypes;<br>Increase satisfaction with the work group;

Session 4	Lisbon – "Empathy"
**Materials and Media**	Cards, pens, box or bag to place cards.
**Strategies/Activities**	
**Introduction**	Summarize the previous session;<br>Reflection on TPP from session 3;<br>Introduce a new theme with *brainstorming* on Empathy;<br>Explain the importance of empathy in daily interactions and how it can improve the group environment;
**Development**	Dynamic 1: "I'm a good observer"<br>Ask the participants to position themselves face to face and to observe each other;<br>Request that half of the participants leave the room to comply with secret instructions; (Indications: change posture (sad, happy, indifferent), hair, tone of voice, clothing, etc.)<br>Return to the initial positions, during (1 min.) and ask "What is different about the environment?"<br>Reflection on dynamics: In our daily lives we are so busy with (distracted) tasks that we stop looking at the other, seeing, hearing/listening whether in more intimate environments or in work contexts... and we don't give the attention we deserve to the other... which could bring problems.<br><br>Dynamic 2: "Perceptions"<br>Distribute 1 sheet and 1 pen to each participant and ask them to draw: "A large animal, small eyes, long tail, protruding ears, big feet" and hairy";<br>Place the participants in a circle sharing the drawings with everyone for 1 min.<br>Reflection on the drawings: How did the same information lead to all different drawings?<br>Reflection on the dynamics: We all have our perception of the world, which is different from the other, through previous life experiences, through our values. Understanding the other's point of view is a challenge within the context of human coexistence.<br>Understand that the way we see the world is not unique and is not always the most accurate.

**Session 4**	**Lisbon – "Empathy"**
	To develop empathy, we need to mentally put ourselves in the other's shoes, which implies opening the mind to the beliefs, ideas, feelings, and attitudes of the other, even if they initially seem foreign to us. This implies that we sometimes put our beliefs and points of view aside, which can be difficult for some people.<br><br>Expository and demonstrative method:<br>Concept of empathy: film viewing: Empathy;<br>Types of empathy;<br>Movie viewing: empathy vs sympathy;<br>Benefits of empathy in interpersonal relationships and in the team environment;<br><br>Realization of the "Circle of Stories" dynamic:<br>Distribute paper cards and pens to each participant;<br>Asking each participant to anonymously write a brief description of a challenging or meaningful experience they have had could be work-related.<br>Collect all the cards and put them in a box or bag;<br>Each participant, in turn, takes a card from the box and reads the experience aloud to the group, suggesting the participants to verbalize how they would feel if they had lived this experience;<br><br>Group discussion after all cards have been read:<br>What was it like to listen to the experiences of others and what did they feel during the activity? Discuss the importance of recognizing and validating the experience of the other, even when they are different from one's own experiences.
**Conclusion**	Summary and feedback of ideas;<br>Completion of the satisfaction questionnaire for session 4.
**Work to think**	Encourage participants to practice empathy during the coming week: by being more attentive, being more observant and practicing tips learned.

Session 4	Lisbon – "Empathy"

Fonte: Elaborate Investigators (2025)

Table S5. Session plan 5 - Algarve – "Emotional management in groups"

**Session 5**	**Algarve – "Emotional management in groups"**
**Duration**	90 minutes
**Objectives**	Develop communication and conflict resolution skills;<br>Identify communication styles;<br>Analyze communication situations, identifying possible assertive responses;<br>Train assertive communication techniques;<br>Increase satisfaction with the work group;<br>Fill in assessment instruments.
**Materials and Media**	Large room, printed questionnaire on communication styles, 3 Cards with different work scenarios that involve challenging emotional situations; Strategy: *Role Play*
**Strategies/Activities**	

Session 5	Algarve – "Emotional management in groups"
**Introduction**	Summarize previous session;<br>Reflection on TPP from session 4;<br>Introduction of a new theme.
**Development**	Expository and demonstrative method:<br>Concept of group relationship management and social skills;<br>Viewing of the film "Acting by emotion";<br>Concept of conflict, types and levels;<br>Tips for conflict management;<br>Communication as a tool for conflict management;<br>Communication styles.<br><br>(Ask participants to fill out an individual questionnaire about the predominant communication style)<br><br>Assertive communication: advantages;<br>Assertiveness training;<br><br>Realization of *Role Play*:<br><br>Request 3 pairs of volunteers to perform *role-play*. Distribute a card with a scenario about an emotionally challenging situation/conflict at work;<br>Each group enacts the situation through *role-play*, showing how each character would react emotionally and how they would deal with the situation;<br>The remaining elements take notes on reactions and strategies used;<br>After each staging, there is room for discussion:<br>- What were the emotions observed in the situation (attention to non-verbal language)?<br>How did each character deal with their emotions and those of the other?<br>- Were the strategies effective? Which could be improved?

Session 5	Algarve – "Emotional management in groups"
	- Encourage constructive *feedback* from participants, highlighting positive points, suggesting improvements;<br><br>Dramatic game:<br>Choose a bus part and through the position of your own body, in a group, build the car;<br>Put the "bus" in motion;<br>Question the participants, who want to, try another piece (explain why they wanted to change and which piece was chosen) and put the "bus" back in motion.<br>In semicircle: individual sharing and group reflection on the dynamics.<br><br>Program closure:<br>Completion of the satisfaction questionnaire for session 5;<br>Completion of a questionnaire on satisfaction and adequacy of the BEmotions psychoeducational intervention *;*<br><br>Completion of the assessment instruments: Veiga Emotional Competence Scale – short version (EVCEr33) (VEIGA-BRANCO, 2023), Satisfaction with the Working Group Scale (DIMAS, 2018).
**Conclusion**	Ask participants for suggestions for improving the program;<br>Thank the participants for their participation, closing the program.

Fonte: Elaborate Investigators (2025)
